# Role of Thermodynamics and Kinetics in the Composition of Ternary III-V Nanowires

**DOI:** 10.3390/nano10122553

**Published:** 2020-12-18

**Authors:** Egor D. Leshchenko, Jonas Johansson

**Affiliations:** Solid State Physics and NanoLund, Lund University, PO Box 118, SE-221 00 Lund, Sweden; jonas.johansson@ftf.lth.se

**Keywords:** composition, ternary nanowires, quaternary liquid melts, Au-catalyzed, modelling

## Abstract

We explain the composition of ternary nanowires nucleating from a quaternary liquid melt. The model we derive describes the evolution of the solid composition from the nucleated-limited composition to the kinetic one. The effect of the growth temperature, group V concentration and Au/III concentration ratio on the solid-liquid dependence is studied. It has been shown that the solid composition increases with increasing temperature and Au concentration in the droplet at the fixed In/Ga concentration ratio. The model does not depend on the site of nucleation and the geometry of monolayer growth and is applicable for nucleation and growth on a facet with finite radius. The case of a steady-state (or final) solid composition is considered and discussed separately. While the nucleation-limited liquid-solid composition dependence contains the miscibility gap at relevant temperatures for growth of In*_x_*Ga_1−*x*_As NWs, the miscibility gap may be suppressed completely in the steady-state growth regime at high supersaturation. The theoretical results are compared with available experimental data via the combination of the here described solid-liquid and a simple kinetic liquid-vapor model.

## 1. Introduction

Considering the current level of technology, one route to further progress is the design and application of nanostructured materials with controlled functional properties. In this context, tuning the composition of ternary III-V semiconductor nanowires (NWs) has been extensively explored [1]. A tremendous number of possible materials have opened up attractive opportunities for the control of their physical properties and enabled the fabrication of materials combinations difficult to achieve in bulk. One important class of such materials are ternary alloy nanowires, which consist of two binary semiconductors that have the same cation or anion. These NWs exhibit unique properties and can be used for specific applications such as InAs_1−*x*_Sb*_x_* [2] and InAs_1−*x*_P*_x_* [3] NW-based infrared photodetectors for air pollution monitoring, GaAs_1−*x*_Sb*_x_* applications in telecommunications industry [4], GaAs_1−*x*_P*_x_* tandem solar cells [5] and Al*_x_*Ga_1−*x*_N nanowire-based mid-deep ultraviolet light emitting diodes [6]. Among nanowires containing different III*_x_*III_1−*x*_V and IIIV_1−*x*_V*_x_* materials, the composition control of In*_x_*Ga_1−*x*_As, GaAs_1−*x*_Sb*_x_* and InAs_1−*x*_Sb*_x_* NWs is the best studied (more than a dozen papers for each materials system [1]). The majority of NWs have been synthesized by both metal organic chemical vapor deposition (MOCVD) and molecular beam epitaxy (MBE) methods via the vapor-liquid-solid mechanism [7] owing to its versatility and possibility to control the NW composition [8], crystal structure [9], morphology [10], growth direction [11], twinning [12] and kinking [13]. At the same time, there is a lack of experimental data on composition control in GaSb_1−*x*_P*_x_* [14], InP_1−*x*_Sb*_x_* [15] and Al*_x_*In_1−*x*_Sb [16] NWs.

It has been shown experimentally that the solid composition of a large number of ternary solid solutions can be varied over the entire range [1]. For some of the materials systems this requires sophisticated growth tricks such as a two-step technique [4] or growth on stems [17]. However, from a thermodynamics point of view, not all the solid compositions can be achieved. Under some conditions the formation of domains consisting of more or less pure binary compounds becomes energetically more favorable. The region in a phase diagram where the formation of a homogeneous ternary solid solution is thermodynamically forbidden is called the miscibility gap and has been observed in structures of different dimensions.

Phase separation in ternary solid solution-based devices results in degradation of their optoelectronic properties, including the inability to extend their operating wavelength to the required values. From this perspective, studies on circumventing the miscibility gap in ternary solid solutions [18] seem very promising. The possible explanations are the formation of metastable states [19], size-dependent effects [20], and the effect of elastic stress which is more relevant for thin films [21]. Moreover, phase separation has a significant effect on the composition of heterointerfaces in axial heterostructured NWs. In theory, for the case of heterostructured NWs, a pronounced miscibility gap could lead to the formation of atomically sharp axial heterojunctions over a wide range of solid composition [22]. However, compositionally graded axial heterointerfaces are observed in experiments [23,24] which might be explained by the reservoir effect [23].

Modelling the solid composition of ternary NWs is often based on rate-limiting steps using kinetic or thermodynamic approaches. In the first one, the solid composition is determined by materials balance equations and can be found from the ratio of the incorporation rates for the two binary species in the steady-state regime [25,26,27]. In this case, the simplest solid-liquid or solid-vapor relationship is given by a one-parametric function [27,28]. The second approach is based on pure thermodynamics and involves the consideration of the formation energy of the nucleus and does not include time-dependent terms. So, the composition in nucleation-limited NW growth is given by a saddle point corresponding to a maximum of the formation energy in the nucleus size and a minimum in its composition [22,29]. It has been shown [22,30] that in this case, the interactions between the components in the liquid phase requires the prevalence of one element over another in the liquid in order to tune the solid composition in a wide range.

Here we present a detailed study of the composition of ternary NWs forming from a quaternary liquid melt combining special cases and providing a complete picture of ternary NW composition evolution. We consider the temporal evolution of the solid composition and the steady-state (or final) composition and their dependence on growth parameters such as temperature, the group V concentration and the Au/III concentration ratio for different materials systems.

A direct comparison of the obtained theoretical liquid-solid composition dependence with experimental data is not possible at the moment. In order to circumvent this, we combine the solid-liquid model with a simple liquid-vapour model which allows us to compare theoretical results with available experimental data. The presented model is useful for the description of the composition of ternary nanowires and can be used in modelling of heterointerface [22] and crystal structure of ternary NWs [31].

## 2. Model

**Figure 1:**
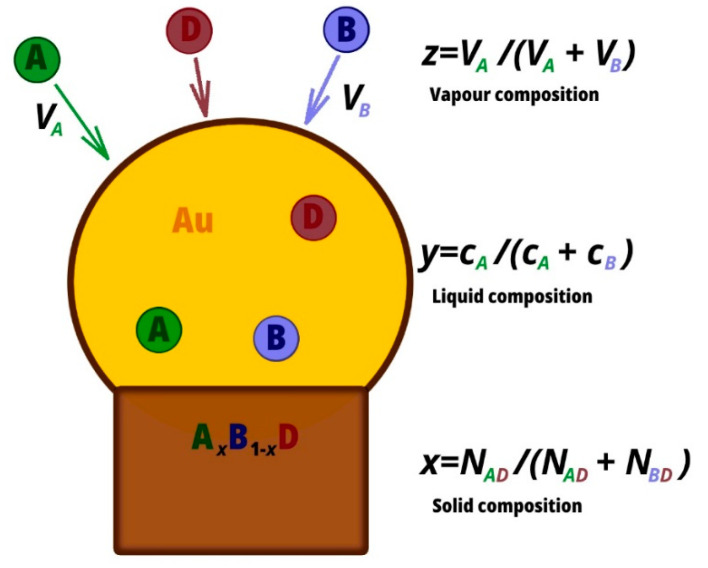
Schematic illustration of the system.

In this paper we study the nucleation of A*_x_*B_1−*x*_D nuclei and their subsequent growth from a quaternary liquid droplet containing A, B, D, and U, where U is a solvent Au, for instance (see Figure 1). Under regular MOCVD and MBE growth conditions, the time between two nucleation events is greater than the time to compete a monolayer which ensures monocenter nucleation and layer-by-layer growth. We make the simplifying assumption that nucleation and monolayer growth occur at a constant composition of the liquid. The solid composition is defined by $x=N_{AD}/\left(N_{AD}+N_{BD}\right)$ where $N_{AD}$ and $N_{BD}$ are the numbers of AD and BD pairs in the monolayer, respectively. The incorporation rates of AD and BD species into the monolayer (or, into the supercritical nucleus) is the differences of respective attachment $W_{k}$ (k=AD,BD) and detachment rates and using detailed balance; these are given by [25,32]:

(1) $$\frac{dN_{AD}}{dt}\;=\;W_{AD}\left(1\;-\;e^{-\Delta \mu_{AD}}\right),$$

(2) $$\frac{dN_{BD}}{dt}\;=\;W_{BD}\left(1\;-\;e^{-\Delta \mu_{BD}}\right).$$

$\Delta \mu_{AD}$ and $\Delta \mu_{BD}$ are the chemical potential differences between the respective species in the liquid phase and the solid phase. The chemical potentials in the case of nucleation of ternary islands from a quaternary liquid melt are well described in [30]. It is clearly seen that at high chemical potential difference ($\Delta \mu_{k}\gg 1$) the bracket term in Equations (1) and (2) tends to 1 and incorporation, and thus composition, are determined by the “kinetic” parameter $W_{k}$. Thus, it stands to reason that the solid composition can be described by a kinetic model under such conditions. At sufficiently low chemical potential difference ($\Delta \mu_{k}\approx 0$) the bracket term has significant influence on the incorporation rate. We assume that the attachment rate is proportional to the concentration of *A* or *B* and *D* elements in the droplet ($c_{A}$ or $c_{B}$ and $c_{D}$) [27] and their diffusivities. The *AD* and *BD* species can incorporate only at the monolayer sidewalls which are in contact with the liquid droplet. Thus, the general form of the attachment rates can be written down as

(3) $$W_{AD}\;=\;f\left(s\right)K_{AD}c_{A}c_{D},$$

(4) $$W_{BD}\;=\;f\left(s\right)K_{BD}c_{B}c_{D}.$$

with $K_{AD}$ and $K_{BD}$ being the incorporation coefficients which depend on the element diffusivities and f(s) is the part of the circumference of the nucleus which is in contact with the liquid droplet. Because of the finite size of a NW, f(s) is a non-monotonic function of the surface coverage [33], and thus, of the nucleus size $s\;=\;N_{AD}\;+\;N_{BD}$. So, in the first stage after nucleation, the effective perimeter increases. When the surface coverage exceeds ~0.5, the monolayer configuration changes abruptly and the effective perimeter after reaching its maximum value decreases down to 0.

The time evolution of the nucleus size is

(5) $$\frac{ds}{dt}\;=\;\frac{dN_{AD}}{dt}\;+\;\frac{dN_{BD}}{dt}$$

Differentiation of the solid composition $x\;=\;N_{AD}/\left(N_{AD}\;+\;N_{BD}\right)$ with respect to time t gives the time evolution of the nucleus composition:

(6) $$\frac{dx}{dt}\;=\;\frac{1}{s}\left(\frac{dN_{AD}}{dt}\;-\;x\left(\frac{dN_{AD}}{dt}\;+\;\frac{dN_{BD}}{dt}\right)\right).$$

Dividing Equation (6) by Equation (5) and eliminating growth time, one obtains

(7) $$\frac{dx}{ds}\;=\;\frac{1}{s}\left(\frac{\;dN_{AD}/dt}{dN_{AD}/dt\;+\;dN_{BD}/dt}\;-\;x\right).$$

We start the analysis by finding the time-invariant steady-state composition of the solid. The case of a steady-state solid composition corresponds to the equality dx/ds = 0 which is satisfied if the bracket term in Equation (7) is zero. Under the circumstances, the steady-state solid composition $x_{s}$ is defined by the incorporation rates $x_{s}\;=\;\left(dN_{AD}/dt\right)/\left(dN_{AD}/dt\;+\;dN_{BD}/dt\right)$. Substituting Equations (1) and (2), the steady-state solid composition is given by

(8) $$x_{s}\;=\;\frac{1}{1\;+\;\frac{1\;-\;y}{y}\frac{K_{BD}}{K_{AD}}\frac{\left(1\;-\;e^{-\Delta \mu_{BD}}\right)}{\left(1\;-\;e^{-\Delta \mu_{AD}}\right)}}.$$

Here the substitution $c_{B}/c_{A}\;=\;\left(1\;-\;y\right)/y$ is used, $y\;=\;c_{A}/\left(c_{A}\;+\;c_{B}\right)$ is the liquid group III composition. Equation (8) is similar to the one from [25] but only with the difference of the proportionality of the attachment rates to the elemental concentrations in the liquid. Moreover, if $\Delta \mu_{AD}\gg 1$ and $\Delta \mu_{BD}\gg 1$, the solid composition is given by the one-parametric equation of $x_{s}\;=\;y/\left(y\;+\;K\left(1\;-\;y\right)\right)$ with $K=K_{BD}/K_{AD}$ which is the same as in [28].

Here we continue to investigate the situation where the composition of the growing monolayer changes with time (dx/ds≠0). So, one may assume that in the beginning the composition of the critical nucleus is completely determined by the chemical potentials whereas later on it is defined by kinetics, the diffusivity ratio, for instance.

Substitution of Equations (1) and (2) in Equation (7) and separation of variables results in

(9) $$\frac{dx}{1/\left(1\;+\;\frac{1\;-\;y}{y}\frac{K_{BD}}{K_{AD}}\frac{\left(1\;-\;e^{-\Delta \mu_{BD}}\right)}{\left(1\;-\;e^{-\Delta \mu_{AD}}\right)}\right)\;-\;x}\;=\;\frac{ds}{s}.$$

It should be noticed that the term f(s) vanishes, which means that the solid composition does not depend on the geometry of the growth and the model is applicable for the finite radius case.

As we mentioned above Equations (1) and (2), these equations describe the incorporation of the AD and BD pairs into supercritical nuclei. The composition and size of the critical nucleus are given by the saddle point of the surface of the nucleus formation energy. Assuming composition independent surface energy term, the x-coordinate of the saddle point can be found from the equality ∂Δμ/∂x = 0 (which is equivalent to $\mu_{i}\;=\;\mu_{j}$ due to the Gibbs–Duhem relation). The nucleation limited composition $x_{*}$ of ternary NWs forming from a quaternary liquid melt was studied in [30] and is given by

(10) $$x_{*}\;=\;\frac{1}{1\;+\;\frac{1\;-\;y}{y}e^{-2\omega_{s}\left(x_{*}-1/2\right)-b}}.$$

Here $\omega_{s}$ is the AD-BD pseudobinary interaction parameter and b is a *y*-dependent parameter whose form can be found in [30].

Because of the presence of the term $\left(1-e^{-\Delta \mu_{BD}}\right)/\left(1-e^{-\Delta \mu_{AD}}\right)$ where $\Delta \mu_{AD}$ and $\Delta \mu_{BD}$ are x-dependent, integration of Equation (9) can only be performed using numerical methods. Anyway, irrespective of its initial value, the solid composition will tend to a stable steady-state value defined by Equation (8). However, one may assume x-independence of $\Delta \mu_{AD}$ and $\Delta \mu_{BD}$ during the integration or at least its weaker influence on the final result than that of 1/(const−x). This approximation is better the smaller $\left|\Delta \mu_{AD}-\Delta \mu_{BD}\right|$ is. Then integration of Equation (9) with the initial condition $x\left(s\;=\;s_{*}\right)\;=\;x_{*}$ with $s_{*}$ being the critical nucleus size gives

(11) $$\frac{x_{appr}\;-\;x_{s}}{x_{*}\;-\;x_{s}}\;=\;\frac{s_{*}}{s}.$$

By substituting $\delta \;=\;s_{*}/s$, Equation (11) can be re-written in the form

(12) $$x_{appr}\;=\;\delta x_{*}\;+\;\left(1\;-\;\delta \right)x_{s}.$$

Equation (12) shows that the solid composition is a linear combination of the steady state and nucleation limited solid compositions. The coefficient δ varies from 1 for the critical nucleus size towards 0 when the size is much larger than the critical nucleus size. Therefore, at δ=1 the solid composition equals the nucleation limited composition while at δ=0 the solid composition coincides with the steady state one. We note that Equation (12) is an approximation and considering the x-dependence of $\Delta \mu_{AD}$ and $\Delta \mu_{BD}$ will lead to a slightly different route to the steady-state composition.

In the case of high supersaturation ($\Delta \mu_{AD}\gg 1$ and $\Delta \mu_{BD}\gg 1$) Equation (11) is the exact solution to Equation (9) with $x_{s}\;=\;x_{kin}$ where

(13) $$x_{kin}\;=\;\frac{1}{1\;+\;\frac{1\;-\;y}{y}\frac{K_{BD}}{K_{AD}}}..\;$$

Within the calculations we assume that the nucleation and growth occur at the same conditions, particularly, at the same liquid compositions. In reality, there are periodic fluctuations in the liquid concentrations during growth. So, after nucleation, atoms of the group V elements in the droplet are consumed quickly to complete the monolayer, after that its concentration restores and the cycle repeats. If this is the case, the liquid-solid composition dependence can be simulated within the developed model if the dependence of the group V concentration on the nucleus size is known (from experiment or by model fitting).

At the moment, the direct comparison of the obtained theoretical results, namely the liquid-solid composition dependence, with experimental data is hampered because of the unknown liquid composition during growth. Thus, while in-situ measurements of the quaternary liquid droplet are not available, ex-situ methods, the most common being energy dispersive X-ray spectroscopy (EDX) are used to measure the final composition. However, the composition of the droplet measured after the NW growth can differ from the liquid composition during the NW growth. Moreover, the primary experimental control parameters are the precursor fluxes, while the liquid composition is an intermediate state and is not so important to know for NW growers. Thus it is necessary to link the solid composition to the vapour composition. In order to do so, let’s consider the equations which describe the materials balance in the droplet [34]:

(14) $$\frac{dN_{A}}{dt}\;=\;V_{A}^{tot}\;-\;\frac{N_{A}}{\tau_{A}}\;-\;\frac{dN_{AD}}{dt},\;$$

(15) $$\frac{dN_{B}}{dt}\;=\;V_{B}^{tot}\;-\;\frac{N_{B}}{\tau_{B}\;}\;-\;\frac{dN_{BD}}{dt}.$$

Equations (14) and (15) describe the change in the number of A and B atoms in liquid ($N_{A}$ and $N_{B}$), respectively: number of atoms increases due to direct impingement of atoms on the droplet and diffusion (the first term) and decreases as a result of desorption (the second term) and the NW growth (the last term). The first terms correspond to atomic influx and consist of direct impingement and surface diffusion fluxes $V_{A}^{tot}\;=\;V_{A}\left(\chi_{A}\;+\;\varphi_{A}\lambda_{A}/R\right)$ and $V_{B}^{tot}\;=\;V_{B}\left(\chi_{B}\;+\;\varphi_{B}\lambda_{B}/R\right)$, where $\chi_{A}$, $\varphi_{A}$, $\chi_{B}$ and $\varphi_{B}$ are vapor-liquid-solid specific coefficients [26], $V_{A}$ and $V_{B}$ are atomic fluxes, R is NW radius and $\lambda_{A}$ and $\lambda_{B}$ are diffusion lengths. The second terms describe desorption from the droplet which depends on adsorption life times $\tau_{A}$ and $\tau_{B}$. Finally, the third terms correspond to decrease of elements due to their incorporation into the monolayers.

In order to simplify the analysis, let us consider the stationary case $dN_{A}/dt\;=\;dN_{B}/dt\;=\;0$. Group V elements are highly volatile which leads to high desorption flux and small diffusion contribution $V_{A}\varphi_{A}\lambda_{A}/R$. Therefore, assuming that desorption is dominating, the number of elements is given by

(16) $$N_{A}\;=\;\chi_{A}V_{A}\tau_{A}$$

(17) $$N_{B}\;=\;\chi_{B}V_{B}\tau_{B}.$$

Introducing the vapor composition $z\;=\;V_{A}/\left(V_{A}\;+\;V_{B}\right)$, division of Equation (17) by Equation (16) gives

(18) $$\frac{1\;-\;y}{y}\;=\;\frac{1\;-\;z}{z}\gamma_{V},$$

with $\gamma_{V}\;=\;\chi_{B}\tau_{B}/\chi_{A}\tau_{A}$. This form is the most convenient for constitution of the liquid composition because one can combine the γ and K parameters and get a one-parametric solid-vapor dependence.

In the case of III*x*III1-*x*V NWs $\gamma_{III}\;=\;\left(R\chi_{B}\;+\;2\varphi_{B}\lambda_{B}\right)/\left(R\chi_{A}\;+\;2\varphi_{A}\lambda_{A}\right)$ and

(19) $$\frac{1\;-\;y}{y}\;=\;\frac{1\;-\;z}{z}\gamma_{III}.$$

## 3. Results and Discussion

### 3.1. Steady State Composition

**Figure 2:**
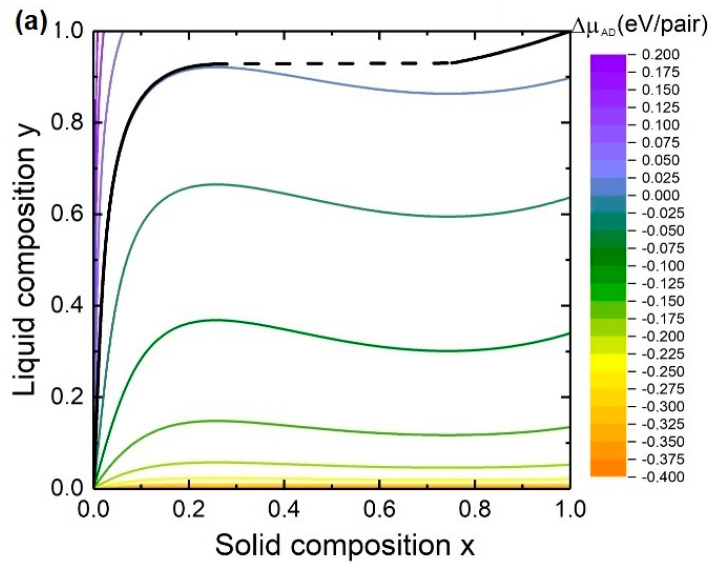

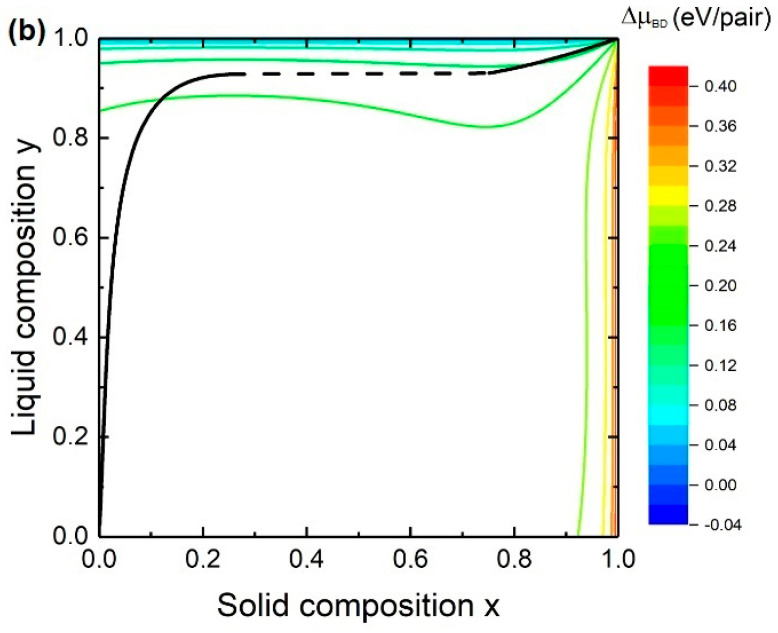
Dependence of the steady-state solid composition on the liquid composition for In*_x_*Ga_1−*x*_As NWs and contour plots of chemical potential differences between the species AD (**a**) and BD (**b**) in the liquid phase and the solid phase. Dashed lines indicate miscibility gaps.

We start the discussion with the consideration of the steady-state composition. Values of the interaction parameters and the Gibbs energies used for calculations here and after are presented in one of our previous papers [30]. Figure 2 shows the liquid-solid composition dependences and the contour plots of chemical potential differences between the species AD (a) and BD (b) in the liquid phase and the solid phase at T=400 °C, $c_{As}\;=\;0.002$, $c_{tot}\;=\;0.9$ and K=1. As can be seen, the y(x) curve follows the lines of $\Delta \mu_{AD}\;=\;0$ at small x and $\Delta \mu_{BD}\approx 0$ at high x. It can be explained by the fact that x≈0 in Equation (8) is satisfied if $\left(1-e^{-\Delta \mu_{AD}}\right)\approx 0$ which means that $\Delta \mu_{AD}\approx 0$. On the other hand, x≈1 is satisfied if $\left(1-e^{-\Delta \mu_{AD}}\right)\approx \left(1\;-\;e^{-\Delta \mu_{AD}}\right)\;+\;\left(1\;-\;e^{-\Delta \mu_{BD}}\right)$ which means that $\Delta \mu_{BD}\approx 0$.

The equality $\Delta \mu_{AD}\;=\;0$ at small solid compositions allows one to find the solid composition from the definition of the chemical potential difference $\Delta \mu_{AD}\;=\;\mu_{A}^{L}\;+\;\mu_{D}^{L}\;-\;\mu_{AD}^{0}\;-\;RTlnx-\omega_{s}{\left(1-x\right)}^{2}$. This simplified solution is given by

(20) $$yc_{tot}=xe^{(\omega_{s}{\left(x-1\right)}^{2}-b_{1})/RT}$$

with $b_{1}\;=\;\left(\mu_{A}^{L}\;-\;RTlnc_{A}\right)+\;\mu_{D}^{L}\;-\;\mu_{AD}^{0}$. However, the equality $\Delta \mu_{BD}\;=\;0$ cannot be used for the calculation of the solid composition at high x because both $\Delta \mu_{BD}$ and $\Delta \mu_{AD}$ have small but finite values.

**Figure 3:**
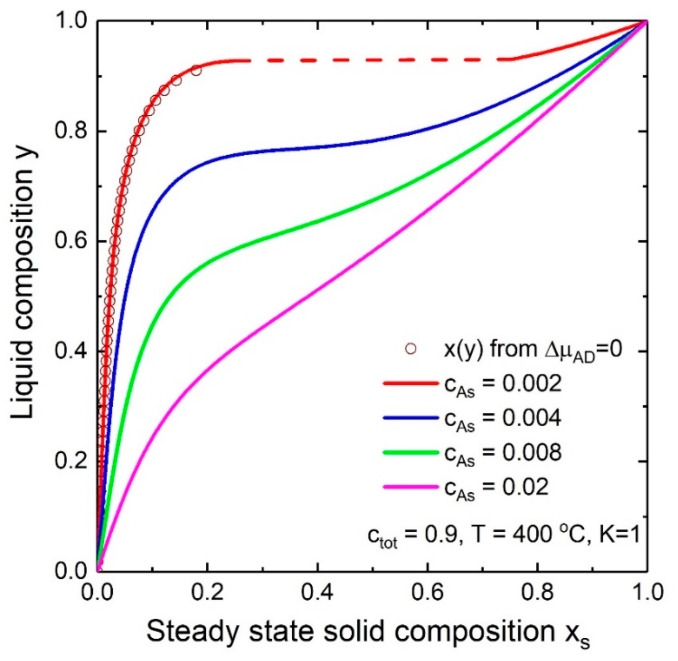
Dependence of the steady-state solid composition on the liquid composition for In*_x_*Ga_1−*x*_As NWs. Solid lines and circles correspond to Equations (8) and (20), respectively. Dashed line indicates miscibility gap.

The liquid-solid composition dependences for In*_x_*Ga_1−*x*_As NWs at T=400 °C, $c_{tot}\equiv c_{A}\;+\;c_{B}\;=\;0.9$ and K = 1 and at different As concentrations are presented in Figure 3. As it is seen, y(x) is highly dependent on the As concentration. This is in contradiction to the nucleation-limited composition where the $y\left(x_{*}\right)$ curve depends on the Au/III concentration ratio and does not depend on the As concentration. The liquid-solid composition curve is similar to the nucleation limited composition dependence at small $c_{As}$ while the shape of the y(x) curve loses its steep behavior with the increase of the As concentration. At relatively high As concentration y(x) becomes more and more linear and approaches y = x. This is due to the strong dependence of the supersaturation on the concentration of group V elements. Moreover, the miscibility gap width reduces with increasing As concentration. Comparing the y(x) curves calculated by Equations (8) and (20) proves that the usage of the simplified solution is appropriate at small solid compositions.

### 3.2. Evolution of the Solid Composition

**Figure 4:**
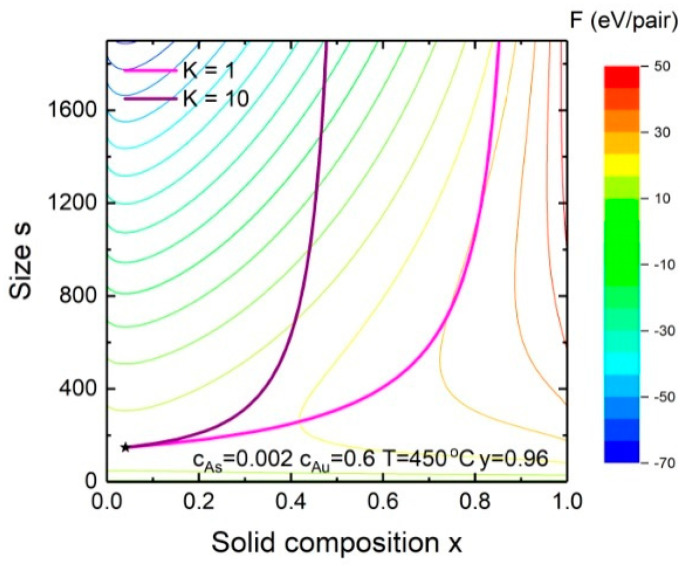
Evolution of the solid composition with nucleus size for In*_x_*Ga_1−*x*_As NWs at y=0.96. The solid thin lines correspond to the levels of the formation energy, *F*.

Next, we analyze the dependence of the solid composition on the nucleus/monolayer size for Au-catalyzed In*_x_*Ga_1−*x*_As NWs. Figure 4 shows the evolution of the solid composition during the vapor-liquid-solid growth of In*_x_*Ga_1−*x*_As NWs at fixed T=450 °C, $c_{As}\;=\;0.002$, $c_{Au}\;=\;0.6$ and y = 0.96 for different K. We do not consider the composition of subcritical nuclei because of their instability and start with the solid composition and size of the critical nucleus. Its position is defined by a saddle point (the star in the lower left corner of Figure 4) where the formation energy (color levels) is at minimum size and maximum composition. After the critical nucleus is formed, the incorporation rates of both species are described by Equations (1) and (2). The solid composition starts to tend to the kinetic composition, “forgetting” its nucleation-limited composition. At a large enough size (~2500) it almost reaches its stable value which is the steady-state composition. Of course, the solid composition might not reach the steady-state composition if a NW radius is very small and the critical size is large. However, according to our estimations, the contribution of nucleation-limited composition in the composition of In*_x_*Ga_1−*x*_As NWs is approximately 5% (δ≈0.05: $s_{*}\approx 200$, s = 4804 for R = 15 nm) under regular MBE and MOCVD conditions. For ternary solid solutions with a high value of the pseudobinary interaction parameter, it might be higher, leading to a significant modification of the final solid-liquid dependence.

**Figure 5:**
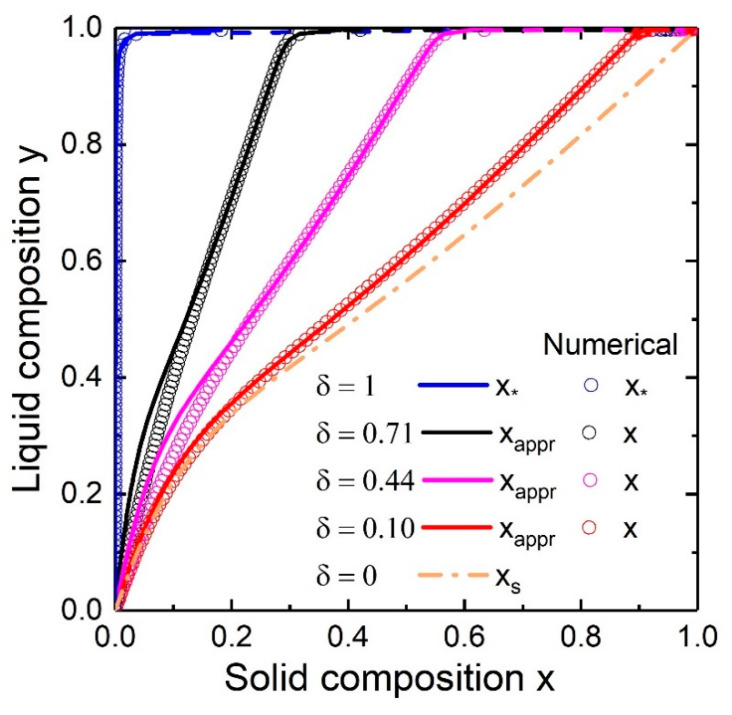
Numerical (circles) and analytical (solid) evolution of the liquid-solid dependence of In*_x_*Ga_1−*x*_As NWs with the size (δ=1, 0.71, 0.44, 0.10, 0) at T=400 °C, $c_{As}\;=\;0.02$ and $c_{Au}\;=\;0.1$. Blue solid and ginger dash-dotted curves correspond to nucleation limited and kinetic composition dependencies respectively. Dashed lines indicate miscibility gaps.

**Figure 6:**
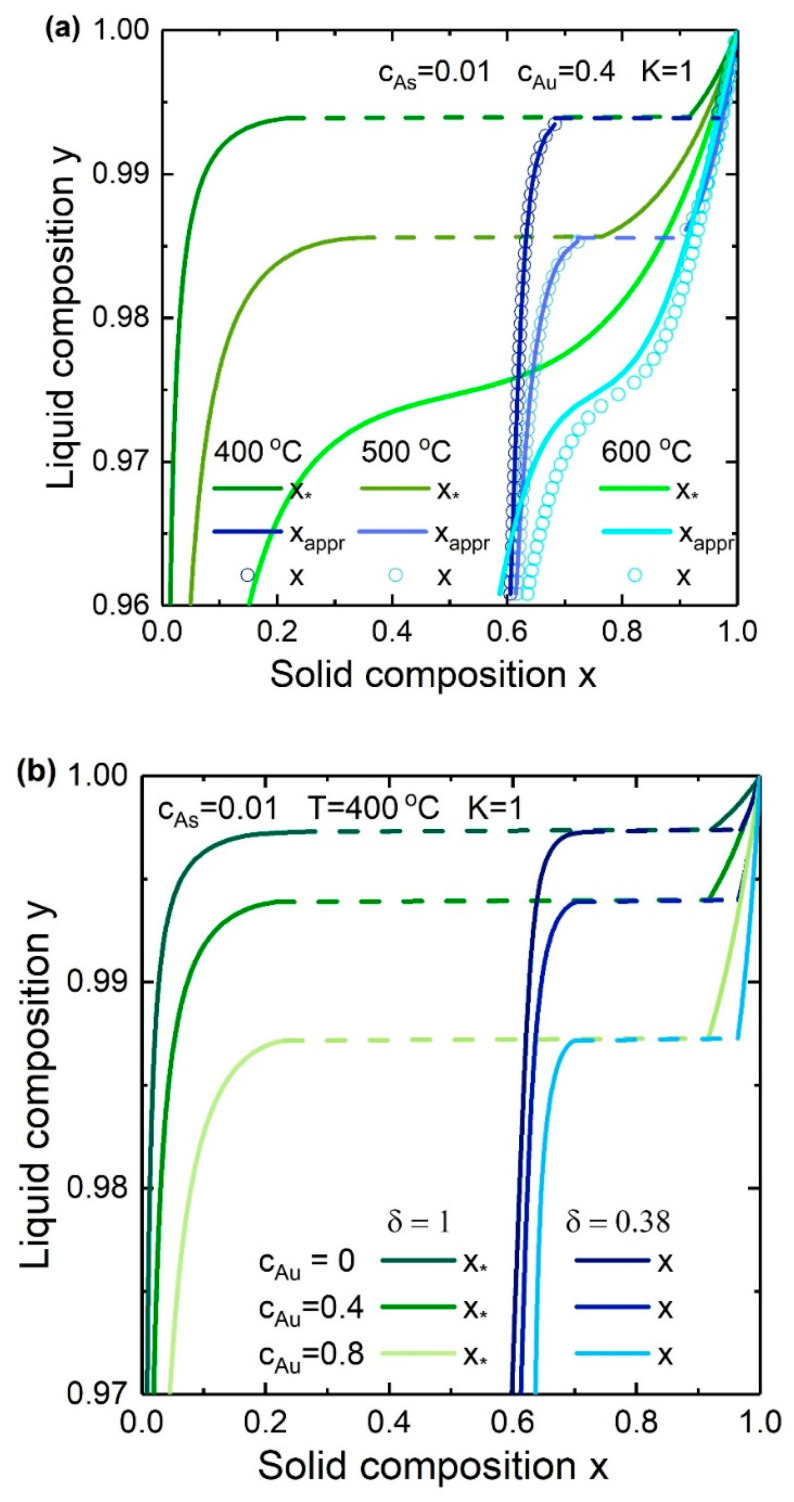
Liquid-solid composition dependences for In*_x_*Ga_1−*x*_As NWs at fixed K=1, $c_{As}=0.01$, δ=0.38, 1 (**a**) for different temperatures at fixed $c_{Au}=0.4$ and (**b**) for different Au/III concentration ratios at fixed T=400 °C.

Figure 5 shows the evolution of the liquid-solid composition dependence for Au-catalyzed In*_x_*Ga_1−*x*_As NWs with the relative nucleus size (δ=1, 0.7, 0.3, 0.05, 0) at T=400 °C, $c_{As}\;=\;0.01$ and $c_{Au}\;=\;0.5$. It can be seen from the curves that the solid composition is a combination of the steady-state (ginger dash-dotted curve) and nucleation-limited (blue curve) solid compositions. Thus, the solid-liquid curve changes from the step-like curve which corresponds to the nucleation-limited composition (δ=1) to the kinetic linear curve of y≈x (δ=0). Moreover, transition from the nucleation limited case to the kinetic case results in a shift and decrease of the miscibility gap: at δ=0.3 the miscibility gap is in the range of 0.73<x<0.96 while at δ=1 it is in the range of 0.12<x<0.88. The parameter K=1 was chosen to reduce the number of model parameters and due to the fact that the diffusivities of group III elements are approximately equal. Its value and the large value of $c_{As}\;=\;0.01$ result in the kinetic liquid-solid curve of y≈x. Approximate analytical solution (Equation (12), solid curves) coincides with the results of numerical integration of Equation (9) (open circles) everywhere except of the little region of small x values. This discrepancy can be explained based on Figure 2: the $1/\left(1-e^{-\Delta \mu_{AD}}\right)$ term in Equation (9) cannot be ignored at small x because $\Delta \mu_{AD}$ along y(x) has a small value (in contrast to $\Delta \mu_{BD}$, which is large) while in the rest of the x range $\Delta \mu_{AD}\approx \Delta \mu_{BD}$ and the condition $\left(1-e^{-\Delta \mu_{BD}}\right)/\left(1-e^{-\Delta \mu_{AD}}\right)\approx 1$ is satisfied. However, at high temperatures of ~600 °C, the discrepancy is sufficiently large and numerical integration is preferable (see Figure 6a).

The influence of the Au/III concentration ratios and temperature on the liquid-solid composition dependence for In*_x_*Ga_1−*x*_As NWs at $c_{As}\;=\;0.01$ and δ = 0.38, 1 is presented in Figure 6. To summarize, the liquid-solid composition dependence follows the same trends as previous results for the nucleation-limited growth [30]. Firstly, the rise in temperature “pushes” the curve down slightly and reduces the miscibility gap width. Secondly, decreasing the Au/III concentration ratio (or, the total group III element concentration) leads to that lower liquid compositions are required to form a certain solid composition at high temperatures. The miscibility gap width stays the same. However, the effect of the Au/III concentration ratio (the shift direction of the y(x) curve) is defined for each system individually. So, for example, the liquid-solid composition dependence for InAs_1−*x*_Sb*_x_* NWs shifts up with increasing the Au/III concentration ratio.

### 3.3. Compositional Limit in Sb-Based Ternary NWs

**Figure 7:**
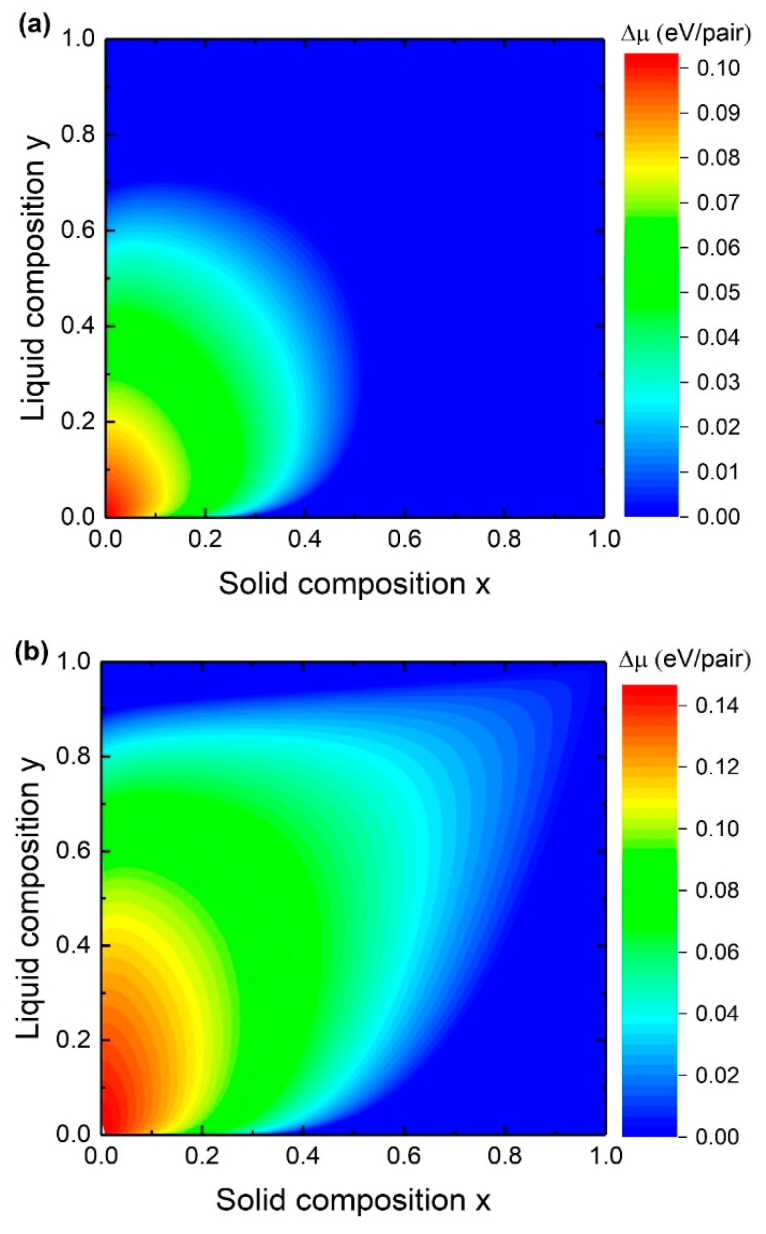

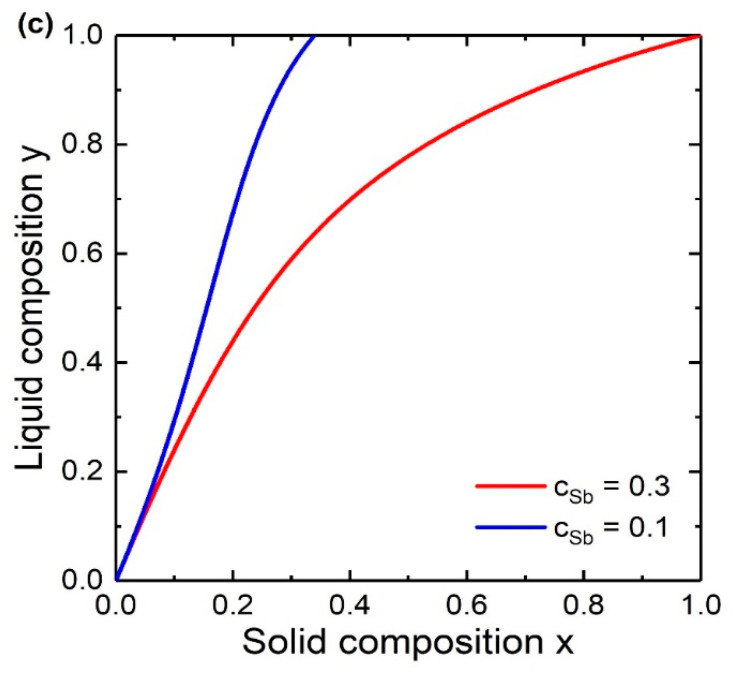
Chemical potential difference between the liquid and solid for different Sb concentrations in the liquid ($c_{Sb}=0.1$ (**a**) and $c_{Sb}=0.3$ (**b**)) and liquid-solid composition dependences of In*_x_*Ga_1−*x*_Sb NWs (**c**) at fixed Au concentration $c_{Au}=0.2$ and temperature T=450 °C. For better clarity, only the positive values of the chemical potential difference are presented. Negative values are shown as level 0 (dark blue).

To explain the composition limit which is observed in experiments [35,36], particularly the difficulty to achieve low (or high) x values in Sb-based ternary alloys, let us consider the chemical potential difference and liquid-solid composition dependences on the example of (Au, In, Ga, Sb) materials system. As seen from liquid-solid composition dependences of Au-catalyzed In*_x_*Ga_1−*x*_Sb NWs (Figure 7c), compositional tuning throughout the entire compositional range is possible in the case of high Sb concentration in the droplet ($c_{Sb}=0.3$) while x>0.35 is forbidden at low supersaturation ($c_{Sb}=0.1$). More fundamentally, low Sb concentration in the droplet leads to a larger area where the chemical potential difference is negative and nucleation is not energetically favourable (which means no NW growth). For example, the liquid-solid composition curve at $c_{Sb}=0.3$ lies inside the area of the positive chemical potential difference (Figure 7b). The area of the positive chemical potential difference is much smaller in the case of low supersaturation (Figure 7a) and limited by range of x<0.4 and y<0.6. Thus, the compositional limit for Sb-based ternary alloys can be explained by the difficulty to get high supersaturation (or, to achieve high concentration of antimony in the droplet). As a comparison, we note that in the case of InGaAs NWs, 1% of As in the droplet is enough to provide a positive chemical potential difference.

### 3.4. Comparison of Theory and Experiment

**Figure 8:**
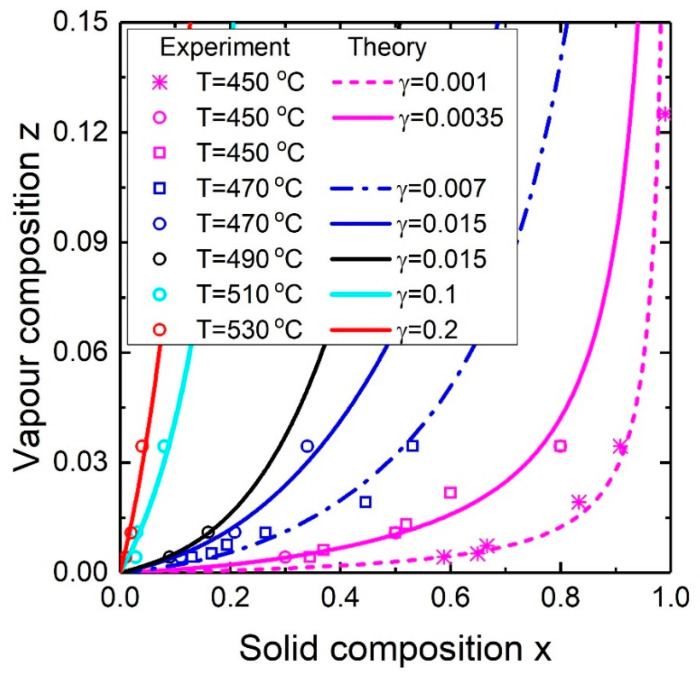
Theoretical (steady-state) and experimental vapor-solid composition dependences for In*_x_*Ga_1−*x*_Sb NWs for different temperatures. The stars represent experimental data from [36]; the rest of the experimental data (squares and circles) are obtained from [35].

As it has been discussed previously, measurement of the liquid concentrations is rarely feasible since it requires special in-situ methods such as EDX in environmental transmission electron microscopy. To verify the developed model, let us combine the steady-state composition model with the presented vapor-liquid model. To be more specific, substitution of Equation (19) into Equation (8) allows to get rid of the term (1−y)/y and calculate the solid composition as a function of the vapor composition. The fitting parameter $\gamma =\gamma_{III}\equiv \left(R\chi_{B}+2\varphi_{B}\lambda_{B}\right)/\left(R\chi_{A}+2\varphi_{A}\lambda_{A}\right)$ defined in the end of the model section shows how efficiently B atoms incorporate into the droplet in comparison with A atoms. Comparison of the theoretical dependence of the ternary NW composition on the vapor composition with the experimental data [35,36] at different temperatures is presented in Figure 8. The only fitting parameter is γ and the fixed parameters are $c_{Sb}=0.2$, $c_{Au}=0.15$ and K=1. As can be seen, the obtained results are in good agreement with experimental data. The change of growth temperature influences the vapor-solid composition curve in an expected manner: a temperature increase shifts the curve to lower solid composition values. So the same γ value has been used to model experimental data at T=490 °C and T=470 °C. However, in the common case, decreasing the temperature from T=530 °C to T=450 °C, the γ value decreases by two orders of magnitude (from γ=0.2 to γ=0.001). Two vapor-solid composition curves at T=450 °C (magenta circles and stars) and two curves at T=470 °C (blue circles and squares) are explained by different Sb fluxes. In our simple model, this is considered by reducing the γ value. The surfactant role of Sb is widely known, and an increase of the Sb flux reduces the In and Ga diffusion lengths, which are included in γ. Another way is to use advanced models in which the group V element flux effects the group III concentration.

## 4. Conclusions

Modelling the solid composition of ternary NWs is of paramount importance for tuning the operating wavelength of NW-based optoelectronic devices and understanding the crystal structure of ternary NWs and heterointerface in NWs. Here we have studied the evolution of the solid composition during ternary NW growth from a quaternary liquid melt using the In*_x_*Ga_1−*x*_As materials system as an example. It has been shown that even if the composition of the critical nucleus is determined by the nucleation-limited regime, the solid composition tends to the “kinetic” steady-state composition. The solid composition is a linear combination of the nucleation-limited solid composition and the kinetic one, whereas the rate of evolution is defined by the ratio of the size of the critical nucleus and the size of the evolving nucleus. While the nucleation-limited liquid-solid composition dependence contains the miscibility gap (at relevant temperatures for the materials systems with high value of the pseudo-binary interaction parameter), the miscibility gap may be suppressed completely in the steady-state growth regime. This can be obtained at high supersaturation as a result of relatively high concentration of group V elements. The solution does not depend on the geometry of the growing nucleus and is applicable in the case of finite NW radius.

The liquid-solid composition dependence (for In*_x_*Ga_1−*x*_As NWs) follows the same trends as in nucleation-limited growth regime: the rise in temperature or Au concentration “pushes” the curve down slightly. It is interesting that the steady-state liquid-solid composition dependence follows the curve of equality to zero of the chemical potential difference of one of the binary components. After considering the chemical potential difference and the liquid-solid composition dependence the compositional limit for Sb-based ternary alloys has been explained. Comparison of theoretical (steady-state) and experimental vapor-solid composition dependences of In*_x_*Ga_1−*x*_Sb NWs from the literature for different temperatures shows good agreement.
